# Composition of *ex vivo* perfusion solutions and kinetics define differential cytokine/chemokine secretion in a porcine cardiac arrest model of lung preservation

**DOI:** 10.3389/fcvm.2023.1245618

**Published:** 2023-09-22

**Authors:** Lena Radomsky, Achim Koch, Carolin Olbertz, Yongjie Liu, Kerstin Beushausen, Jana Keil, Ursula Rauen, Christine S. Falk, Jenny F. Kühne, Markus Kamler

**Affiliations:** ^1^ Institute of Transplant Immunology, Hannover Medical School, Hannover, Germany; ^2^ Department of Thoracic and Cardiovascular Surgery, West German Heart Center, University Hospital Essen, Essen, Germany; ^3^ Institute of Biochemistry, University of Duisburg-Essen, Essen, Germany; ^4^ DZIF, German Center for Infectious Diseases, Germany, TTU-IICH, Hannover—Braunschweig site, Braunschweig, Germany; ^5^ DZL, German Center for Lung Diseases, BREATH site, Hannover, Germany

**Keywords:** graft preservation, *ex vivo* lung perfusion, porcine, Steen solution, Custodiol-N, dextran, albumin, cytokines/chemokines

## Abstract

**Background:**

*Ex vivo* lung perfusion (EVLP) uses continuous normothermic perfusion to reduce ischemic damage and to improve post-transplant outcomes, specifically for marginal donor lungs after the donation after circulatory death. Despite major efforts, the optimal perfusion protocol and the composition of the perfusate in clinical lung transplantation have not been identified. Our study aims to compare the concentration levels of cytokine/chemokine in different perfusion solutions during EVLP, after 1 and 9 h of cold static preservation (CSP) in a porcine cardiac arrest model, and to correlate inflammatory parameters to oxygenation capacities.

**Methods:**

Following cardiac arrest, the lungs were harvested and were categorized into two groups: immediate (I-EVLP) and delayed EVLP (D-EVLP), after 1 and 9 h of CSP, respectively. The D-EVLP lungs were perfused with either Steen or modified Custodiol-N solution containing only dextran (CD) or dextran and albumin (CDA). The cytokine/chemokine levels were analyzed at baseline (0 h) and after 1 and 4 h of EVLP using Luminex-based multiplex assays.

**Results:**

Within 4 h of EVLP, the concentration levels of TNF-α, IL-6, CXCL8, IFN-γ, IL-1α, and IL-1β increased significantly (*P* < 0.05) in all experimental groups. The CD solution contained lower concentration levels of TNF-α, IL-6, CXCL8, IFN-γ, IL-2, IL-12, IL-10, IL-4, IL-1RA, and IL-18 (*P* < 0.05) compared with those of the Steen solution. The concentration levels of all experimental groups have correlated negatively with the oxygenation capacity values (*P* < 0.05). Protein concentration levels did not reach statistical significance for I-EVLP vs. D-EVLP and CD vs. CDA solutions.

**Conclusion:**

In a porcine cardiac arrest model, a longer period of CSP prior to EVLP did not result in an enhanced protein secretion into perfusates. The CD solution reduced the cytokine/chemokine secretion most probably by iron chelators and/or by the protecting effects of dextran. Supplementing with albumin did not further reduce the cytokine/chemokine secretion into perfusates. These findings may help in optimizing the preservation procedure of the lungs, thereby increasing the donor pool of organs.

## 1. Introduction

Lung transplantation (LTX) is the ultimate treatment option for patients suffering from end-stage lung disease. However, there is a huge discrepancy between available donor organs and patients with demand, resulting in high waiting list mortality (1). To overcome this disparity, transplantation centers have extended their selection criteria (2–4), and few countries have legalized the donation after circulatory death (DCD), aside from the donation after brain death (DBD). *Ex vivo* lung perfusion (EVLP) has been discussed as an alternative preservation technique to improve the outcome of marginal or DCD organs. In contrast to the standard procedure, which is based on cold static preservation (CSP) and thus reduced metabolism, the donor organ is perfused with normothermic solution during EVLP (5). Therefore, EVLP reduces the cold ischemic time and the ischemia–reperfusion injury (IRI) of the organ (6). Furthermore, EVLP allows to evaluate the condition of the graft and to therapeutically treat donor organs prior to implantation (7, 8). In experimental studies and human clinical trials, EVLP has shown promising results (9–11). Still, it is not clear whether the donor organ needs to be perfused directly after procurement or if a prolonged CSP prior to perfusion is harmful to the lungs. Moreover, the optimal composition of the perfusion solution in reducing IRI has not been defined.

This study aimed to examine whether immediate EVLP (I-EVLP), after only 1 h of CSP, or delayed EVLP (D-EVLP), after 9 h of CSP, would result in different cytokine/chemokine concentrations in perfusates. We used a porcine model as an established alternative to human LTX, in which we previously demonstrated a comparable lung function of immediate vs. delayed perfusion (12). Moreover, a Custodiol-N solution supplied with dextran was shown to have a positive impact on the functional parameters of the EVLP-preserved porcine lungs (13). In the present study, we investigated the impact of modified Custodiol-N solution on cytokine/chemokine patterns in perfusates and correlated the data to oxygenation capacity measurements collected from the same lungs (12, 13). The differences in cytokine/chemokine concentrations could help identify the ideal start point of perfusion and the optimal composition of the perfusion solution to preserve the DCD lungs with reduced IRI, resulting in improved transplant engraftment, survival, and ultimately increased donor pool.

## 2. Material and methods

### 2.1. Animals

All animals included in this study received human care compliant with the “Principles of Laboratory and Animal Care” and the Guide for the Care and Use of Laboratory Animals, which was composed by the Institute of Laboratory Animal Resources and published by the National Institutes of Health (NH publication no. 86–23, revised 1996). The study only involves organ procurement from animals because none of them underwent medical treatment prior to euthanasia. According to the applicable German law (§1 VTMVO), the study was reported to the local Landesamt für Natur, Umwelt und Verbraucherschutz NRW and supervised by the Central Animal Laboratory of the University Duisburg-Essen.

### 2.2. Chemicals

The compositions of the different perfusate solutions are displayed in Table 1. Steen Solution™ and Perfadex™ were purchased from XVIVO Perfusion (Gothenburg, Sweden). Custodiol-N was acquired from Dr. F. Köhler Chemie (Bensheim, Germany). Pyrogen-free dextran 40 (AppliChem, Darmstadt, Germany) was added to the modified Custodiol-N solution in a concentration of 50 g/L. The D-EVLP with Custodiol-N solution supplied with dextran + albumin (D-EVLP/CDA) solution was additionally supplemented with 7 g/L of bovine serum albumin (Carl Roth, Karlsruhe, Germany). The sterilization process of the solution was performed by filtration using a 0.22 µm filter (Filtropur BT25, Sarstedt, Nümbrecht, Germany). Directly before use, a 10 ml of 10% glucose solution (G-10, B. Braun, Melsungen, Germany) was added to 1 L of the CD and CDA solutions.

**Table 1:** Composition of perfusion solutions.

	CD (Custodiol-N plus dextran)	CDA (Custodiol-N + dextran and albumin)	Steen™
Sodium	16	16	86
Potassium	10	10	4.6
Magnesium	8	8	0.8
Calcium	0.02	0.02	1.5
Chloride	30.04	30.04	
Histidine	124	124	
*N*-Acetylhistidine	57	57	
Sucrose	33	33	
α-Ketoglutarate	2	2	
Aspartate	5	5	
Glycine	10	10	
Alanine	5	5	
Tryptophan	2	2	
Arginine	3	3	
Deferoxamine (μmol/L)	25	25	
LK 614 (μmol/L)	7.5	7.5	
Dextran (g/L)	50	50	5
Albumin (g/L)		7	70
Glucose			11
Phosphate			1.2
pH	7.0^a^	7.0^a^	7.4^b^
Osmolarity (mOsm/L)	306	306	

All concentrations are given in mmol/L unless stated otherwise.

^a^ At 20°C.

^b^ Adjusted to pH 7.4 with sodium hydroxide.

### 2.3. Experimental procedure

Mature domestic pigs (age = 13–15 weeks) were sedated using ketamine (30 mg/kg; i.m.) combined with azaperone (0.05 mg/kg; i.m.), and these pigs were anesthetized afterward with midazolam (0.1 mg/kg; i.v.) and ketamine (0.3 mg/kg; i.v.). This DCD model represents euthanasia without previous treatment for lung protection because the pigs were neither heparinized nor ventilated during anesthesia. Sternotomy was performed after cardiac arrest (Maastricht category III) (14), and visible safe signs of death were observed as previously described (5, 15). Warm ischemia time was on average 60 min, and the ensuing lungs were then flushed antegradely and retrogradely with 4 L of 4°C cold low potassium dextran (LPD) solution (Perfadex™; XVIVO Perfusion, Gothenburg, Sweden) added with trometamol (1 mmol/L) and heparin (100 IU/L).

### 2.4. Experimental groups

Through a random selection process, four experimental groups were formed. The lungs were either prepared for the following processes: (i) I-EVLP (*n* = 10) after 1h of technical CSP followed by perfusion, or placement in standard preservation bags for 9h in 1L of 4°C LPD solution and subsequent perfusion (delayed perfusion). For the perfusion of the D-EVLP lungs, different acellular solutions were used: (ii) Steen solution (D-EVLP; *n* = 8) or modified Custodiol-N solution containing either (iii) dextran alone (D-EVLP/CD; *n* = 8) or (iv) dextran and albumin (D-EVLP/CDA; *n* = 8). The Toronto protocol (6) and the perfusion system XPS™ (XVIVO Perfusion, Gothenburg, Sweden) were used to perform 4 h of EVLP on all the four groups (Supplementary Figure S1A).

### 2.5. Cytokine and chemokine quantification

The perfusion solution samples were obtained at the beginning of perfusion (0 h) and after 1 and 4 h of perfusion. The samples were stored at −80°C. To quantify the concentration levels of 13 soluble molecules in the perfusates at the different time points, Luminex-based multiplex assay (Millipore porcine cytokine/chemokine 13-Plex, Merck, Darmstadt, Germany) was used according to the manufacturer's instructions. Standard curves and concentrations were calculated using the Bio-Plex Manager 6.1 software.

### 2.6. Oxygenation capacity measurement

Oxygenation capacity is defined as the difference between the pulmonary arterial pressure and the venous oxygen pressure. In this study, it was measured hourly during EVLP by blood gas analysis of the perfusates (ABL 700, Radiometer, Copenhagen, Denmark) at an FiO_2_ of 1.0.

### 2.7. Statistical analyses

For descriptive statistical analyses, the GraphPad Prism software (version 9, La Jolla, CA, USA) was used. According to the D’Agostino–Pearson omnibus normality testing, the cytokine/chemokine concentrations were not normally distributed. Therefore, nonparametric two-tailed unpaired *t*-test (Kruskal–Wallis test) was used to compare the two groups. When comparing different time points between individuals of the different experimental groups, a paired *t*-test (Wilcoxon test) was used. For correlation analyses, Spearman test and linear regression were applied. Principal component analysis (PCA) plots and heatmaps were generated using the Qlucore Omics Explorer software (version 3.5, Lund, Sweden). Two-group or multigroup comparisons were used to identify cytokines/chemokines that differed most significantly between the experimental groups. Therefore, the data were log_2_ transformed and scaled to mean zero, variable one, and a threshold of 0.001. The statistical test used in each analysis and the *q*-value used as a cut-off are indicated in the figure legends. Significance was considered for *P*-values <0.05.

## 3. Results

To better understand the inflammatory reperfusion response induced during ischemia prior to implantation of the donor lung, we analyzed the cytokine/chemokine pattern in perfusates of the DCD lungs comparing different preservation protocols in a porcine model. The lungs were prepared for (i) immediate EVLP (I-EVLP) with Steen solution, (ii) cold storage for 9 h and subsequent EVLP with Steen solution (D-EVLP), (iii) subsequent EVLP with Custodiol-N solution added with dextran (D-EVLP/CD) alone, or (iv) subsequent EVLP with Custodiol-N solution added with dextran and albumin (D-EVLP/CDA; Table 1, Supplementary Figure S1A).

### 3.1. D-EVLP and I-EVLP samples separate by perfusion time point rather than by CSP duration

**Figure 1:**
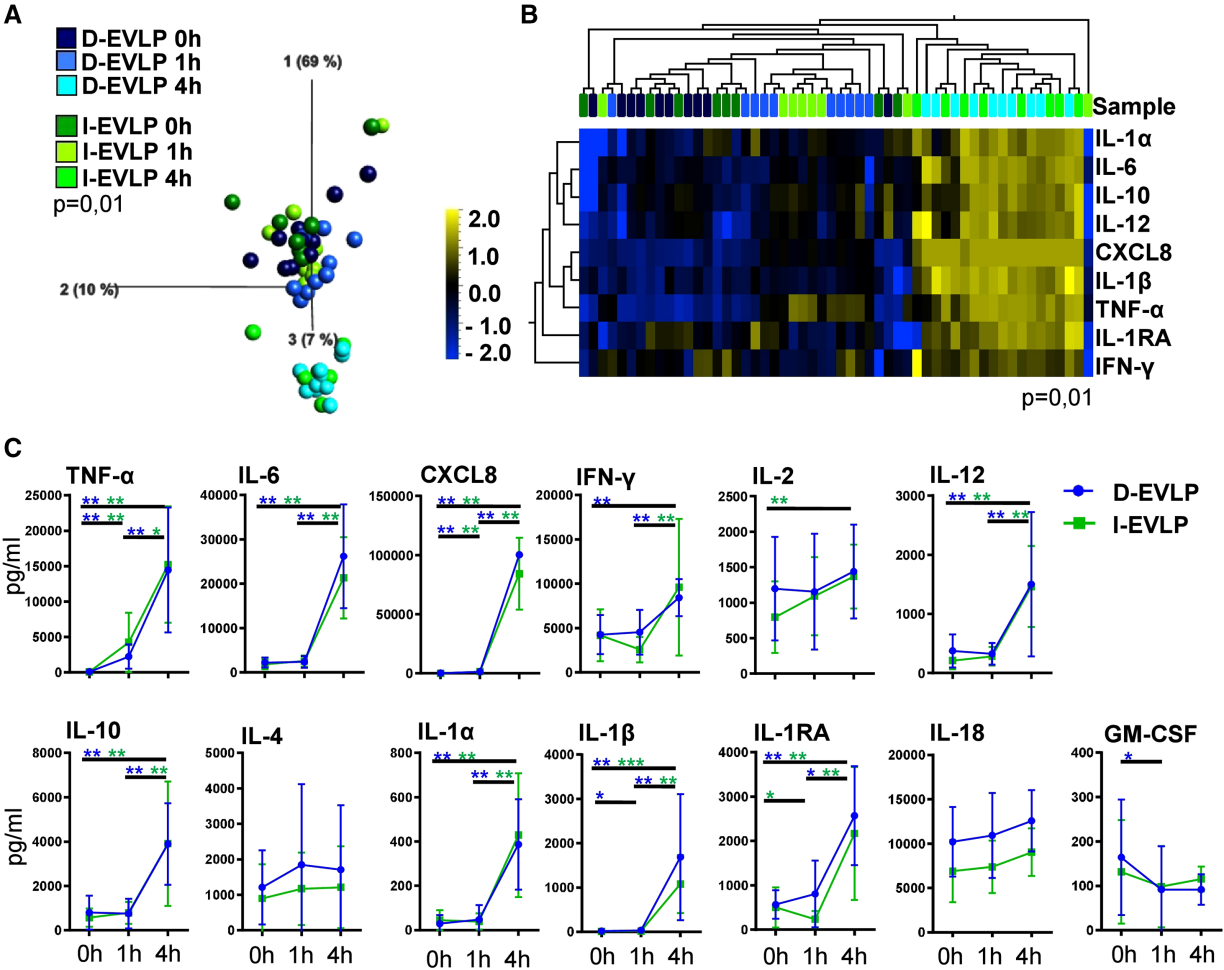
D-EVLP and I-EVLP samples separate by perfusion time point rather than by CSP duration. Perfusion samples were obtained directly at the beginning (0 h) and 1 and 4 h after perfusion and assessed for cytokine/chemokine secretion by Luminex-based multiplex assays. (**A**) Principal component analysis of the 13 cytokines according to delayed or immediate perfusion and time point (*P* = 0.01 and *q* = 0.001) and (**B**) unsupervised hierarchical clustering are shown. Two-group comparisons were used to identify variables differentially expressed between the two groups. Blue color indicates lower expression, and yellow color indicates higher expression. (**C**) Cytokine concentrations are displayed in time response for the two experimental groups. For statistical analysis, the different time points of each group were compared using a paired *t*-test (Wilcoxon). To compare the D-EVLP and I-EVLP groups at the different time points, a two-tailed, unpaired *t*-test (Kruskal–Wallis) was applied. Data are shown as mean ± SEM; asterisks indicate *P*-values with *P < 0.05, **P < 0.01, ***P < 0.001, and ****P < 0.0001.

To analyze for a potential effect of longer CSP times on the cytokine/chemokine pattern in the perfusates, we compared the I-EVLP samples with the D-EVLP samples. Interestingly, we found protein concentrations of the immediate and delayed EVLP samples to divide by perfusion time point rather than by CSP duration in PCA, displaying comparable cytokine/chemokine concentrations in perfusates of the groups using Steen perfusion solution (Figure 1A, Supplementary Figure S1). The samples obtained after 4 h of EVLP clearly separate from the other time points and generally show higher protein concentrations (Figure 1A). Unsupervised hierarchical cluster analyses (UHC) identified nine cytokines/chemokines with significantly different levels in perfusates between the start (0 h) and the end (4 h) of perfusion, namely, the pro-inflammatory cytokines/chemokines, such as TNF-α, IL-6, CXCL8/IL-8, IFN-γ, IL-12, IL-1α, and IL-1β, and IL-10 and IL-1RA, which are known to suppress the inflammatory response (*P* = 0.01; Figure 1B). Perfusates of the D-EVLP and I-EVLP group contained significantly higher concentrations after 4 h of perfusion of typical Th1 cytokines (TNF-α, IFN-γ, IL-12), Th2 cytokine IL-10, pro-inflammatory factors (IL-1α, IL-1β, IL-6, CXCL8), and the regulatory IL-1RA when compared with those found in the samples obtained at the beginning of perfusion (0 h) (INF-γ only for D-EVLP). Of note, TNF-α and CXCL8 levels were already significantly elevated after 1 h of immediate or delayed EVLP (*P* < 0.05). For all other analytes, the main increase in concentration was detected between 1 and 4 h of EVLP (Figure 1C), which indicates an intensified secretion of the proteins by the lung, reflecting the IRI.

### 3.2. D-EVLP/CD samples show a trend toward reduced protein secretion especially after 4 h of perfusion compared with the D-EVLP/CDA group

**Figure 2:**
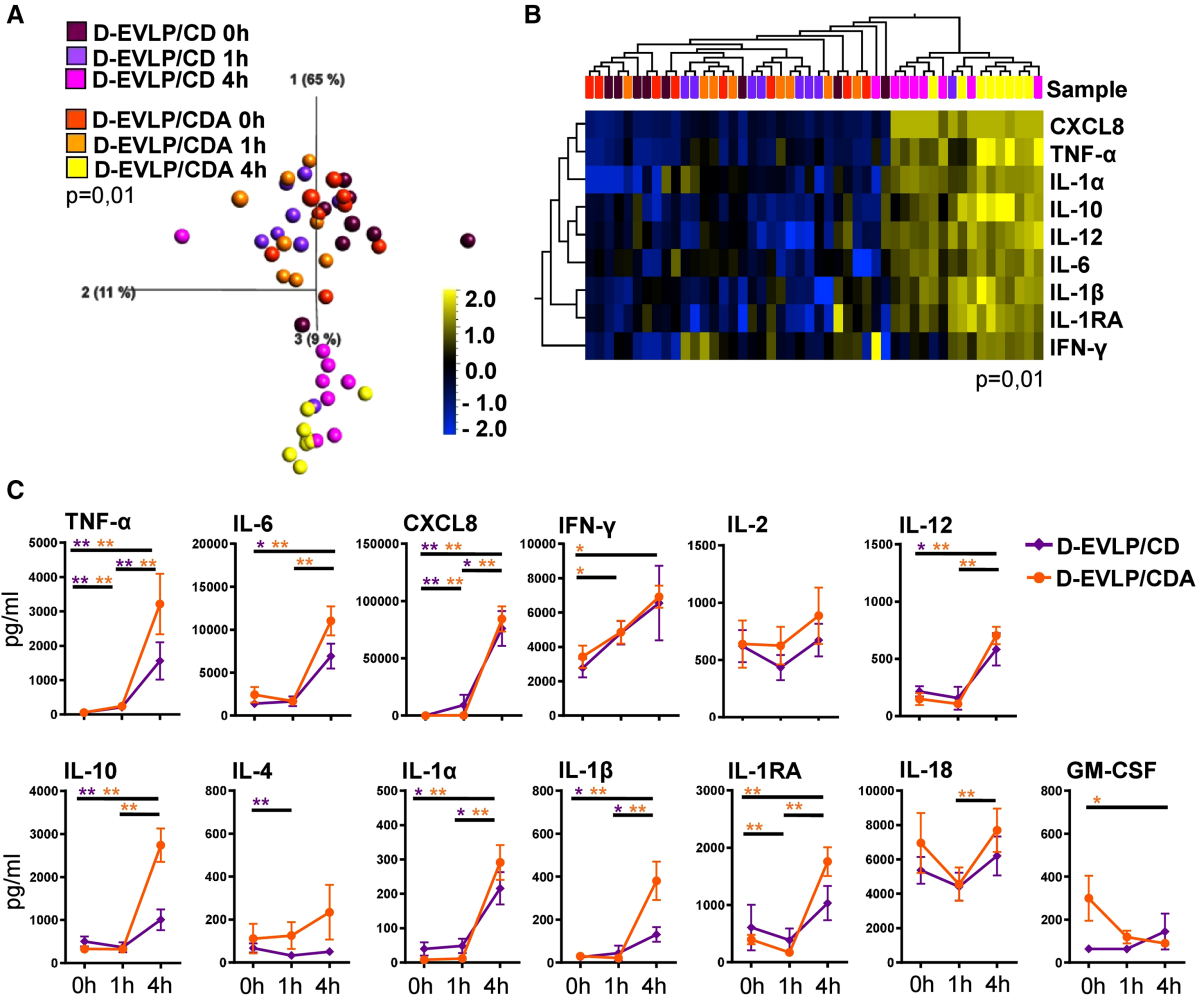
D-EVLP/CD and D-EVLP/CDA samples show separation by time point rather than by adding supplements to the perfusion solution. Samples were obtained and measured as described in Figure 1. (**A**) Principal component analysis of the 13 cytokines according to experimental groups and time point (*P* = 0.01 and *q* = 0.004) and (**B**) unsupervised hierarchical clustering are shown. Two-group comparisons were used to identify variables differentially expressed between the two groups. Blue color indicates lower expression, and yellow color indicates higher expression. (**C**) Cytokine concentrations are displayed in time response for the two experimental groups. For statistical analysis, the different time points of each group were compared using a paired *t*-test (Wilcoxon). To compare the D-EVLP/CD and D-EVLP/CDA groups at the different time points, a two-tailed, unpaired *t*-test (Kruskal–Wallis) was applied. Data are shown as mean ± SEM; asterisks indicate *P*-values with *P < 0.05, **P < 0.01, ***P < 0.001, and ****P < 0.0001.

Elucidating whether albumin in addition to dextran affects the protein secretion of the allografts, we compared the cytokine/chemokine concentrations in D-EVLP/CD and D-EVLP/CDA perfusion samples. The samples were separated by analysis time point in PCA, with a significant increase in concentrations after 4 h of EVLP (Figure 2A). This finding indicates a comparable protein secretion and accumulation in the Custodiol-N groups. In line with this observation, the D-EVLP/CD and D-EVLP/CDA samples were clustered together, when comparing all four experimental groups (Supplementary Figure S1). Eight cytokines/chemokines were identified by UHC with significantly different levels in perfusates at the different sampling time points: the pro-inflammatory TNF-α, IL-6, CXCL8, IFN-γ, IL-12, and IL-1β and the anti-inflammatory IL-10 and IL-1RA (*P* = 0.01; Figure 2B). Interestingly, both UHC and PCA revealed a slight separation of the D-EVLP/CD and D-EVLP/CDA samples after 4 h of perfusion, showing the D-EVLP/CD samples trending toward lower concentrations when compared with the CDA samples.

Comparing the different analysis time points for the D-EVLP/CD and D-EVLP/CDA groups, we found significant differences in protein concentrations for both groups were detected between the 0 and 1 h time points for TNF-α and CXCL8. However, the main increases were observed when comparing the samples obtained at 4 h with the samples obtained at earlier time points. Between 0 h and the end of perfusion (4 h) in both the D-EVLP/CD and D-EVLP/CDA groups, the concentrations of TNF-α, IL-6, CXCL8, IL-10, IL-12, IL-1α, and IL-1β were significantly elevated. An increase of protein concentration comparing 1 and 4 h time points in both groups was measured for TNF-α, CXCL8, IL-1α, and IL-1β. Remarkably, we detected a trend to lower concentrations and less increase in protein concentrations over time in the D-EVLP/CD group compared with the D-EVLP/CDA group. IL-1RA levels did not increase in the D-EVLP/CD group but were significantly elevated at 1 and 4 h in the D-EVLP/CDA group (*P* < 0.01; Figure 2C). These findings suggest less cytokine/chemokine secretion of D-EVLP/CD lungs when compared with the D-EVLP/CDA organs.

In conclusion, the D-EVLP/CD and D-EVLP/CDA samples showed no significant differences at the individual sampling time points, but a trend to lower protein concentrations in the D-EVLP/CD group was observed, indicating that there was no reducing effect of albumin on the protein secretion of the allograft.

### 3.3. Lowest cytokine/chemokine accumulation in the D-EVLP/CD group compared with all other experimental groups

**Figure 3:**
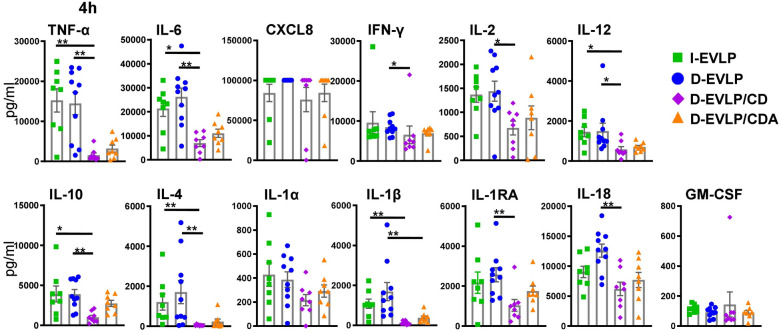
Lowest cytokine/chemokine accumulation in the D-EVLP/CDA group compared with all other experimental groups. The samples were obtained and measured as described in Figure 1. Cytokine/chemokine concentrations in the different perfusion solutions are displayed for each cytokine after 4 h of perfusion. For statistical analyses comparing the concentrations in the different experimental groups, a two-tailed, unpaired *t*-test (Kruskal–Wallis) was applied. Data are shown as mean ± SEM; asterisks indicate *P*-values with *P < 0.05, **P < 0.01, ***P < 0.001, and ****P < 0.0001; non-significant differences were not labeled specifically.

In the next step, we dissected the differences in protein concentrations in the groups at 4 h of EVLP in more detail (Figure 3). The D-EVLP/CD samples reached significantly lower protein concentrations of TNF-α, IL-6, IL-12, IL-10, and IL-4 when compared with those in the delayed and immediate perfusion samples with Steen solution (I-/D-EVLP; *P* < 0.05; Figure 3). IFN-γ, IL-2, and IL-1RA were significantly less concentrated in the D-EVLP/CD samples compared with the D-EVLP (Steen) samples, but not in the I-EVLP samples. IL-1β concentrations displayed a slightly different pattern from the rest by showing significantly lower concentrations in the D-EVLP/CDA samples compared with the D-EVLP samples after 4 h of perfusion. Furthermore, IL-1β levels were significantly reduced in the D-EVLP/CD group compared with the I-EVLP group. Although significantly lower CXCL8 levels were quantified at 0 and 1 h of EVLP in the D-EVLP/CD and D-EVLP/CDA samples compared with Steen solution samples (Supplementary Figures S2A,B), no differences were detected after 4 h of EVLP, as all groups reached the highest measurable concentrations. To highlight the reduced cytokine/chemokine concentration in the D-EVLP/CD and D-EVLP/CDA groups, we performed a relative comparison of these experimental groups with the D-EVLP group. Therefore, the mean values were calculated for each group and time point. Subsequently, the mean values of the D-EVLP group were normalized to 100%, and relative percentages of the other groups were calculated. The most extensive reduction of concentrations after 4 h of perfusion was detected for IL-4 in the D-EVLP/CD group with only 3% of the compared D-EVLP concentrations. Also, IL-1RA, IL-1β, IL-2, IL-6, IL-10, IL-12, IL-18, and TNF-α have shown a reduction of >50% when comparing the D-EVLP/CD group with the D-EVLP group. The cytokine concentrations in perfusates using Custodiol-N solution supplied with dextran and albumin also displayed a relative reduction, but not as pronounced as in the D-EVLP/CD group (Supplementary Figure S3).

These findings suggest a reducing effect of Custodiol-N plus dextran, but not albumin, on the IRI and subsequent cytokine secretion of the allograft, resulting in less protein accumulation in perfusates after 4 h of EVLP.

### 3.4. Negative correlation of oxygenation capacities and cytokine/chemokine concentrations in perfusates

**Figure 4:**
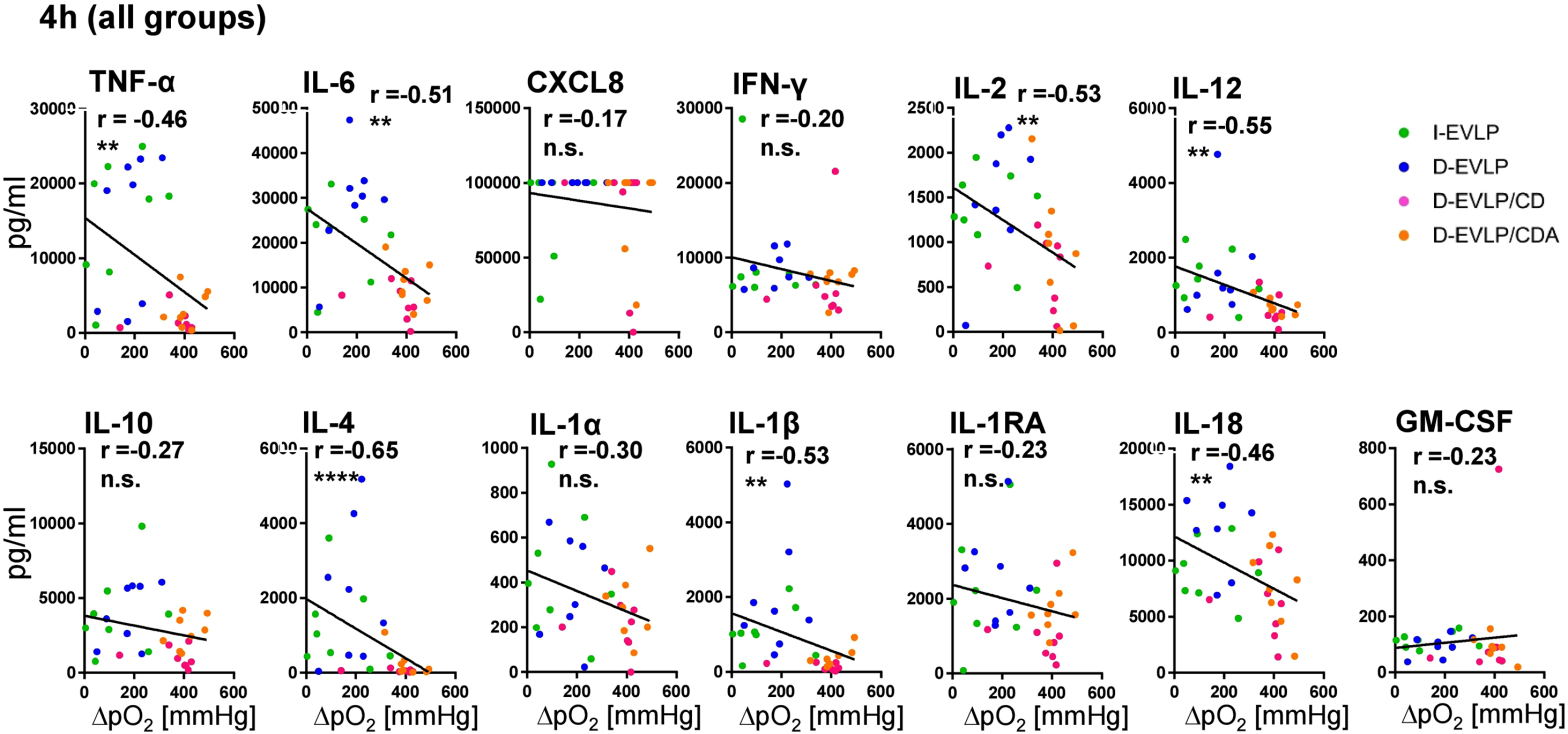
Negative correlations of oxygenation capacity and cytokine/chemokine concentrations in perfusates. Perfusion samples for cytokine/chemokine quantification were obtained and measured as described in Figure 1. Oxygenation capacity (ΔpO_2_) was measured as described in the methods section. The different perfusates were labeled in colors; blue: D-EVLP; green: I-EVLP; pink: D-EVLP/CD; orange: D-EVLP/CDA. Correlation analyses (Spearman) of the oxygenation capacity and cytokine/chemokine concentrations after 4 h of perfusion were calculated. To highlight the negative correlation between these two variables, a linear regression of perfusion samples was performed. Asterisks indicate *P*-values with *P < 0.05, **P < 0.01, ***P < 0.001, and ****P < 0.0001.

In order to reveal the potential effects of the different EVLP solutions on lung function, we correlated oxygenation capacities, measured in perfusates of all groups during EVLP, with the cytokine/chemokine concentrations. After 4 h of EVLP, higher oxygenation capacities were found to significantly correlate with lower concentrations of TNF-α, IL-6, IL-2, IL-12, IL-4, IL-1β, and IL-18 in all four experimental groups (*P* < 0.01; Figure 4). This negative correlation was evident for all measured cytokines/chemokines, except for granulocyte-macrophage colony-stimulating factor (GM-CSF), when including not only the 4 h time point, but also all the time points in the analysis (*P* < 0.05; Supplementary Figure S4). Taken together, these findings confirmed for EVLP to reduce IRI in the donor organs as evidenced by reduced cytokine/chemokine secretion.

## 4. Discussion

*Ex vivo* perfusion is a tool to preserve and improve especially marginal donor organs prior to transplantation. Despite major efforts, the optimal perfusion protocol and the precise composition of the perfusion solution in clinical LTX have not been identified. Our study aimed to investigate whether the CSP prior to EVLP (immediate *vs*. delayed) and the composition of the perfusion solution (Steen solution vs. Custodiol-N solution supplied with dextran or dextran/albumin) have impacts on the cytokine/chemokine secretion of the graft during EVLP and on the inflammation of the donor organ during the preservation process. We made use of an established surrogate porcine LTX model. The protein concentrations in perfusates were quantified at different time points during EVLP using Luminex-based multiplex assays and were then correlated to oxygenation capacities (12, 13).

We did not observe significant differences in the protein concentrations in perfusates (0 h) when comparing immediate and delayed EVLP after 1 and 9 h of CSP, respectively, indicating no further ischemic injury induced due to longer period of CSP. Interestingly, also after 1 and 4 h of perfusion, the protein concentrations in perfusates of the D-EVLP and I-EVLP samples remained comparable.

Consequently, longer period of CSP prior to EVLP seemed not to impact the cytokine/chemokine secretion of the graft during reperfusion. This is in line with previous findings, demonstrating that the pulmonary function of the harvested pig lungs was not impaired after a prolonged CSP when compared with immediate perfusion (12, 16). Of the analyzed proteins, only IL-18 showed a trend toward higher concentrations in perfusates of the D-EVLP samples, although not reaching significance. Similar observations were made in a porcine liver transplant model with elevated IL-18 concentrations in perfusate after a prolonged CSP (17), and IL-18 enhances the secretion of pro-inflammatory IFN-γ (18, 19). Remarkably, we did not detect increased IFN-γ concentrations in the delayed perfusion samples, suggesting that longer period of CSP in the D-EVLP lungs did not translate into an enhanced secretion of inflammatory mediators (i.e., IFN-γ). Consequently, delayed *ex vivo* perfusion (9 h CSP) might represent a feasible strategy of lung preservation.

In addition, there could be beneficial effects of hypothermia on the donor organs, not only via suppression of pro-inflammatory mediators but also via enhanced secretion of tissue-protective cytokines such as IL-22 (20, 21). Moreover, lower expression of GSK-3β and reduced activation of NF-κB, two key players of the inflammatory cascade, were demonstrated in a rat model of hemorrhagic shock (22–24). Recent research focused on controlled hypothermia with stable temperatures of approximately 4°C–10°C to prevent freezing of the tissue. In this regard, it was shown that for heart and lung TX, the organs preserved with controlled hypothermia not only showed a normal perioperative function but also showed a reduced severity of primary graft dysfunction (21, 25, 26). However, our results have shown neither positive nor negative effects of longer period of CSP on the cytokine concentrations in perfusates.

Aside from the cold injury, the lung allograft is damaged upon reperfusion during EVLP and after implantation in the recipient not only through the inflammatory cascade involving immune cells but also through the response of both endothelial and epithelial cells. It was shown in several studies and in different organs that epithelial cells, as well as endothelial cells, are capable of secreting cytokines and chemokines after stimulation with danger signals or pro-inflammatory mediators (27, 28).

As a result of the ongoing IRI in the graft, cytokines/chemokines likely accumulate during 4 h of perfusion. Due to augmenting concentrations over time, the first hours of reperfusion rather than the duration of CSP seem crucial for the inflammation of the donor organ. Consequently, the aim is to optimize the reperfusion process, resulting in a reduced IRI. However, the analysis of the reperfusion upon implantation of the perfused donor organ was beyond the scope of our study. Although in a porcine model of LTX, lower cytokine/chemokine concentrations in perfusates (through EVLP combined with cytokine absorption) lead to an improved organ function even after TX (29).

Aside from the CSP duration, the chemical composition of the perfusion solutions could also affect the reperfusion process, hence potentially causing inflammation of the graft. The standard perfusion solution for EVLP is acellular Steen solution containing human serum albumin. However, in recent years, several alternative perfusion solutions have been developed, such as Custodiol-N, a modified cardioplegic solution designed for cold storage of solid organs (30–33). In contrast to the Steen solution, Custodiol-N contains the iron chelators deferoxamine and LK614, binding redox-active ions that might otherwise lead to the production of highly reactive oxygen species and subsequently lead to graft injury. A central role of iron ions in cold-induced cell injury (consisting of hypothermic injury and subsequent rewarming injury) and the protective effects of iron chelators were demonstrated in diverse cell types, including hepatocytes, endothelial cells, and cultured lung epithelial cells (34–36). Based on these observations, we hypothesize that Custodiol-N reduces the cytokine/chemokine secretion of the allograft during EVLP when compared with Steen solution, thereby reducing the inflammatory response of the transplanted graft. Therefore, another experimental group perfused with Custodiol-N, supplemented with Dextran40 (D-EVLP/CD) (33, 37), was included in our study. Along with maintaining colloid osmotic pressure, Dextran40 is known for its anti-thrombotic features and for protecting the endothelium from excessive leukocyte interactions by coating the endothelial surface (38). Finally, the perfusion solution of the fourth experimental group in our study was additionally supplemented with albumin (D-EVLP/CDA), which has been shown to stabilize and protect the endothelial glycocalyx by contributing to the endothelial surface layer (39).

Comparing cytokine/chemokine concentrations in perfusates, we observed significant differences depending on the perfusion solutions used. The groups employing Steen solution displayed higher concentrations of nearly all measured proteins when compared with groups perfused with Custodiol-N + Dextran40, with the difference being most pronounced after 4 h of perfusion. Endothelial protection by deferoxamine and LK614 possibly reduces the inflammatory reperfusion response in the lungs, although protection by other components of the solution and the protective effects on lung epithelial cells might also contribute to reducing inflammatory response. Moreover, lower concentrations of cytokine/chemokine could be the result of dextran inhibiting leukocyte interactions by coating and thereby protecting the endothelium. Yet, in the present study, it was not possible to assess the secretion of endothelial adhesion molecules (i.e., porcine intercellular adhesion molecule (ICAM) and vascular cell adhesion molecule (VCAM)) in the perfusates of the different experimental groups.

The beneficial effects of albumin on edema formation during heart perfusion were shown in a porcine study (40). Thus, we conceived that albumin could have a positive effect on the inflammation of the lungs by protecting the glycocalyx during the rewarming period. Contrary to our expectations, the addition of albumin to the perfusion solution had no reducing effect on cytokine/chemokine secretion during 4 h of EVLP since we did not detect significant differences in cytokine/chemokine concentrations at any sampling time point when comparing the D-EVLP/CD and D-EVLP/CDA groups. However, the D-EVLP/CD group showed a trend toward lower concentrations of the measured proteins. We lately reported slightly improved oxygenation capacities of pig lungs after 4 h of EVLP when perfused with Custodiol-N solution supplemented with dextran and albumin when compared with the addition of dextran only (13). The beneficial effects of albumin during prolonged period of EVLP might be due to improved oncotic support as evidenced by somewhat lower wet/dry weight ratios (13). Superior lung function in our cohort could result from the reduced inflammation due to elevated anti-inflammatory mediators (i.e., IL-10, IL-1RA) in the D-EVLP/CDA group.

Recently, it was shown that higher cytokine/chemokine levels in serum (i.e., IL-6, CXCL8) after transplantation are associated with worse clinical outcomes (41, 42). The correlation of the cytokine/chemokine concentrations with the oxygenation capacities of the lungs after 4 h of EVLP in our study revealed higher protein concentrations in perfusates to correlate with worse lung function. In line with our findings, it was shown that lower concentrations of pro-inflammatory IL-1β in perfusates are associated with better lung function 24 h post-transplantation (43). Moreover, cytokine absorption prior to LTX resulted in an improved lung function, decreased inflammation, and less primary graft dysfunction in a porcine model, underlining the importance of reducing cytokine and chemokine concentrations during preservation (29, 44, 45). Therefore, D-EVLP/CD would provide the most usable donor organs for transplantation by reaching the lowest concentrations of cytokine/chemokine in the perfusate.

In our experimental groups, the D-EVLP/CD samples contained the lowest protein concentrations of e.g. the pro-inflammatory cytokines/chemokines CXCL8 and IL-6, known to be involved in the pathogenesis of primary graft dysfunction, and the elevated levels of CXCL8 in perfusates were suggested to predict the risk of the primary graft dysfunction (42, 46). Interestingly, the concentrations of the anti-inflammatory cytokines IL-10 and IL-1RA were also reduced in the D-EVLP/CD perfusate samples, suggesting a decreased secretion of pro- and anti-inflammatory mediators in the D-EVLP/CD group. Hence, the suppression of immune cells with hypothermia will reduce not only the pro-inflammatory cytokine secretion from these cells but also the anti-inflammatory cytokine secretion (47).

## 5. Conclusion

A prolonged CSP (9 h) does not affect the cold injury of the lung, while the first hours of reperfusion seem to be crucial for the damage of the allograft. Moreover, Custodiol-N supplied with dextran showed a reducing effect on the cytokine/chemokine secretion, while albumin exhibited no additional effect during 4 h of EVLP. The cytokine/chemokine concentrations in perfusates of Custodiol-N solution supplied with dextran and albumin negatively correlated with the oxygenation capacities, suggesting their usage as an alternative perfusion solution to the standard Steen solution. Possibly, by using Custodiol-N solution with dextran and albumin, more organs would reach the clinically relevant threshold of oxygenation capacity of >350 mmHg, and thus the transplant volume could be increased. Along with the composition of perfusate solutions, cytokine/chemokine concentrations in perfusates can be lowered by applying cytokine absorption filters. The latter were demonstrated to improve lung function of transplanted organs due to reduced IRI (29, 44, 45).

## 6. Limitations

The limitation of our study is focusing on the *ex vivo* perfusion period. The lungs were not transplanted, and there were no available data with regard to the post-perfusion period. Moreover, we only captured the oxygenation capacities as data displaying the lung function of the grafts.

## Data Availability

The raw data supporting the conclusions of this article will be made available by the authors, without undue reservation.
