# Supplementary material for: Composition of ex vivo perfusion solutions and kinetics define differential cytokine/chemokine secretion in a porcine cardiac arrest model of lung preservation
Source: Front Cardiovasc Med. 2023 Sep 22;10:1245618. doi: 10.3389/fcvm.2023.1245618 (PMC10556242; doi:10.3389/fcvm.2023.1245618)
Supplement: Supplementary file 1 [file Datasheet1.pdf]

Supplementary Material

Composition of ex vivo perfusion solutions and kinetics define differential cytokine/chemokine secretion in a porcine cardiac arrest model of lung preservation

Lena Radomsky, Achim Koch, Carolin Olbertz, Yongjie Liu, Kerstin Beushausen, Jana Keil, Ursula Rauen, Christine S. Falk, Jenny F. Kühne*, Markus Kamler

*** Correspondence:** Jenny F. Kühne: Kuehne.Jenny@mh-hannover.de

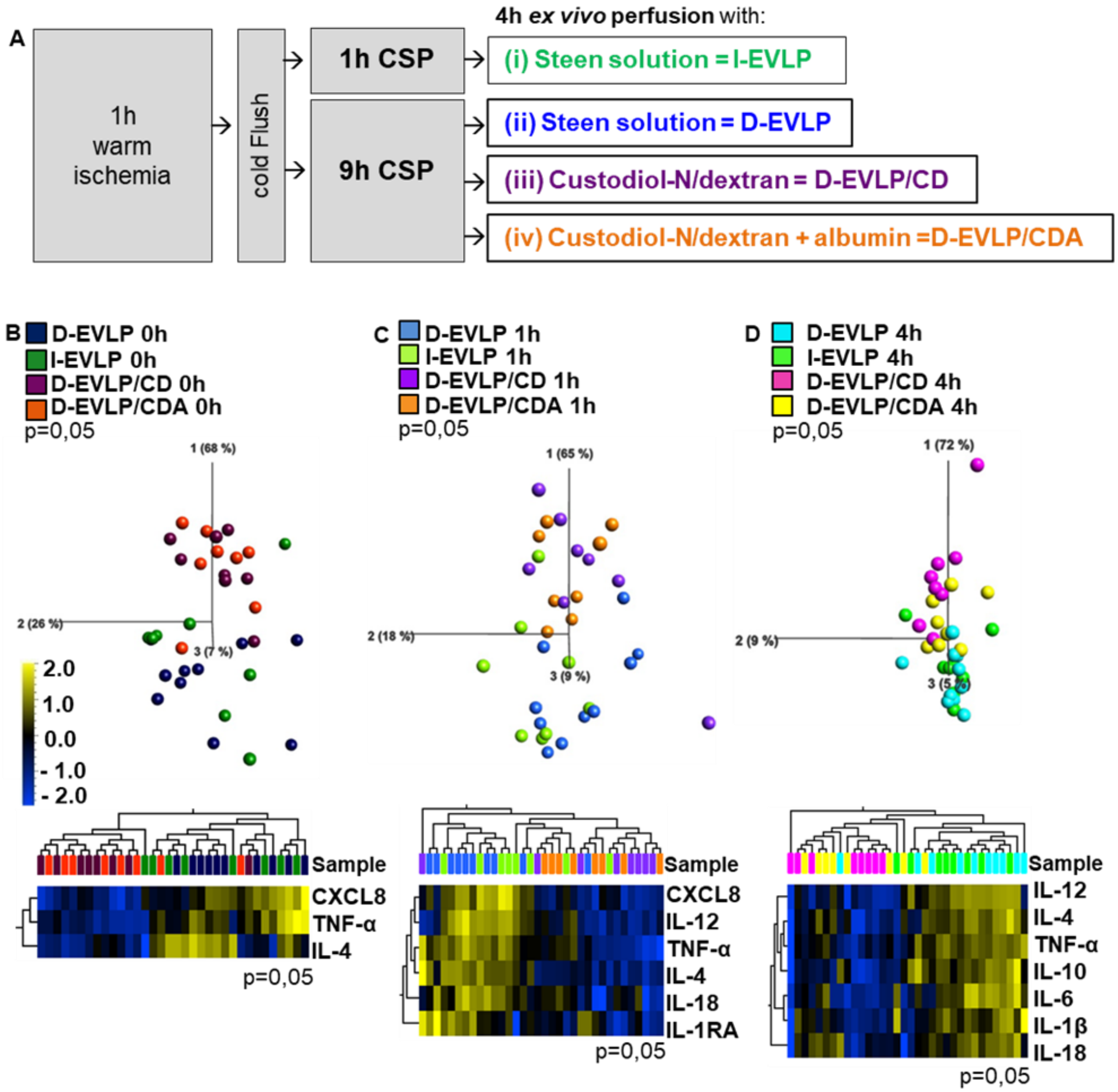

Supplementary Figure 1. Principal component and unsupervised cluster analyses groups D-EVLP and I-EVLP samples together and separates D-EVLP/CD and D-EVLP/CDA samples. Perfusion samples for cytokine/chemokine quantification were obtained and measured as described in Fig.1. Protein concentrations were analyzed according to experimental groups at (A) beginning of perfusion (0h) (P=0.05 and q= 0.018), (B) 1h of perfusion (P=0.05 and q=0.043) and (C) 4h of perfusion (P= 0.05 and q=0.022). Principal component analyses of the 13 soluble analytes and unsupervised hierarchical clustering are shown. Multiple group comparisons were used to identify variables differentially expressed between the subgroups. Blue color indicates lower, yellow color indicates higher expression. D-EVLP, delayed ex vivo perfusion with Steen solution; I-EVLP, immediate ex vivo perfusion with Steen solution; D-EVLP/CD, delayed ex vivo perfusion with Custodiol-N solution containing dextran; D-EVL/CDA, delayed ex vivo perfusion with Custodiol-N solution containing dextran and albumin.

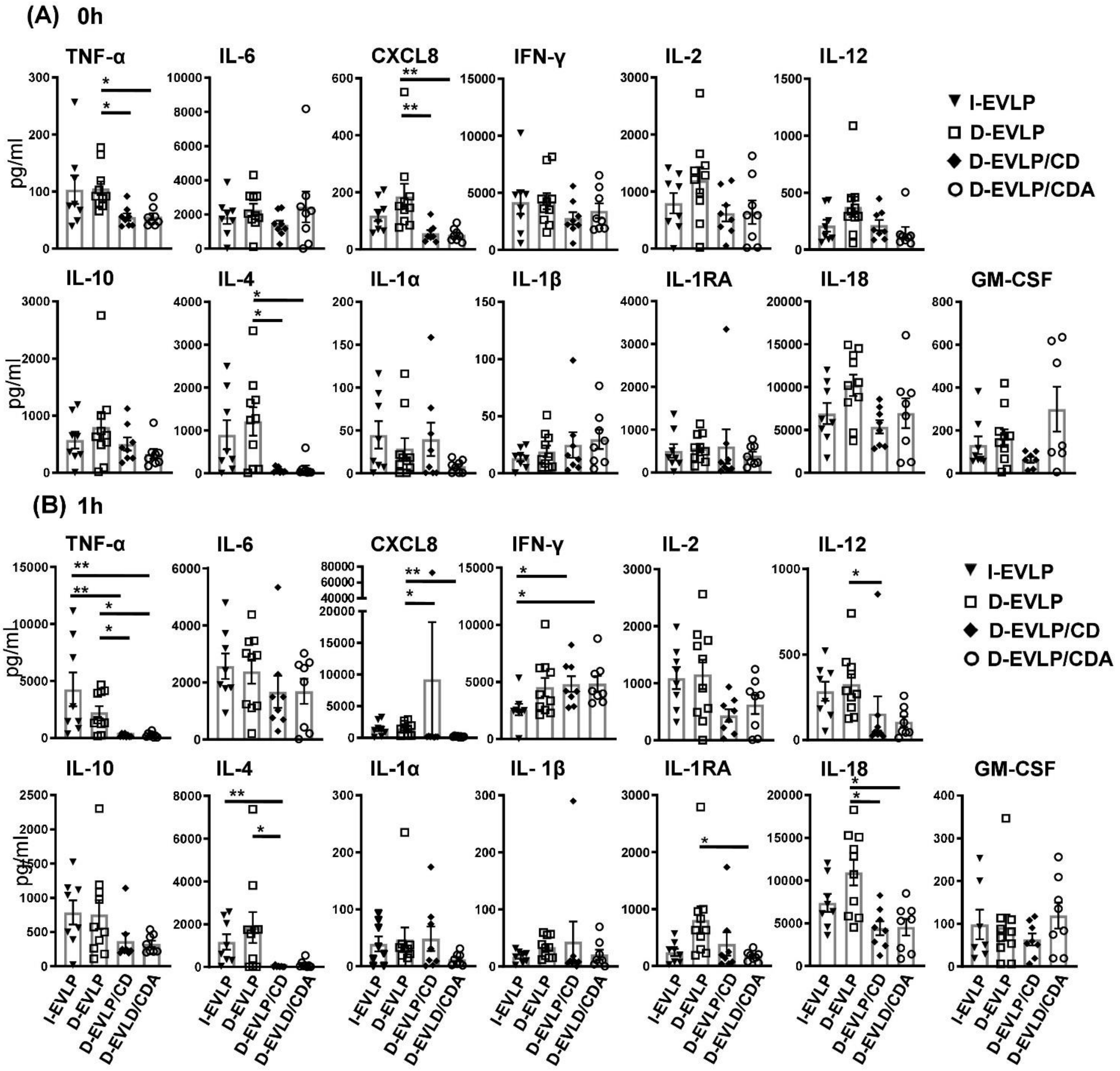

Supplementary Figure 2. Significant differences in cytokine/ chemokine concentrations after 0h and 1h of perfusion between the four experimental groups. Samples were obtained and measured as described in Fig. 1. Cytokine/chemokine concentrations in the different perfusion solutions are displayed for each cytokine after (A) beginning of perfusion and (B) 1h after perfusion. For statistical analyses comparing the concentrations in the different experimental groups, a two-tailed, unpaired t-test (Kruskal-Wallis) was applied. Data are shown as mean ± SEM, asterisks indicate P values with $*P < .05$ $**P < .01$, $***P < .001$, $****P < .0001$, non-significant differences were not labeled specifically. D-EVLP, delayed ex vivo perfusion with Steen solution; I-EVLP, immediate ex vivo perfusion with Steen solution; D-EVLP/CD, delayed ex vivo perfusion with Custodiol-N solution containing dextran; D-EVLP/CDA, delayed ex vivo perfusion with Custodiol-N solution containing dextran and albumin.

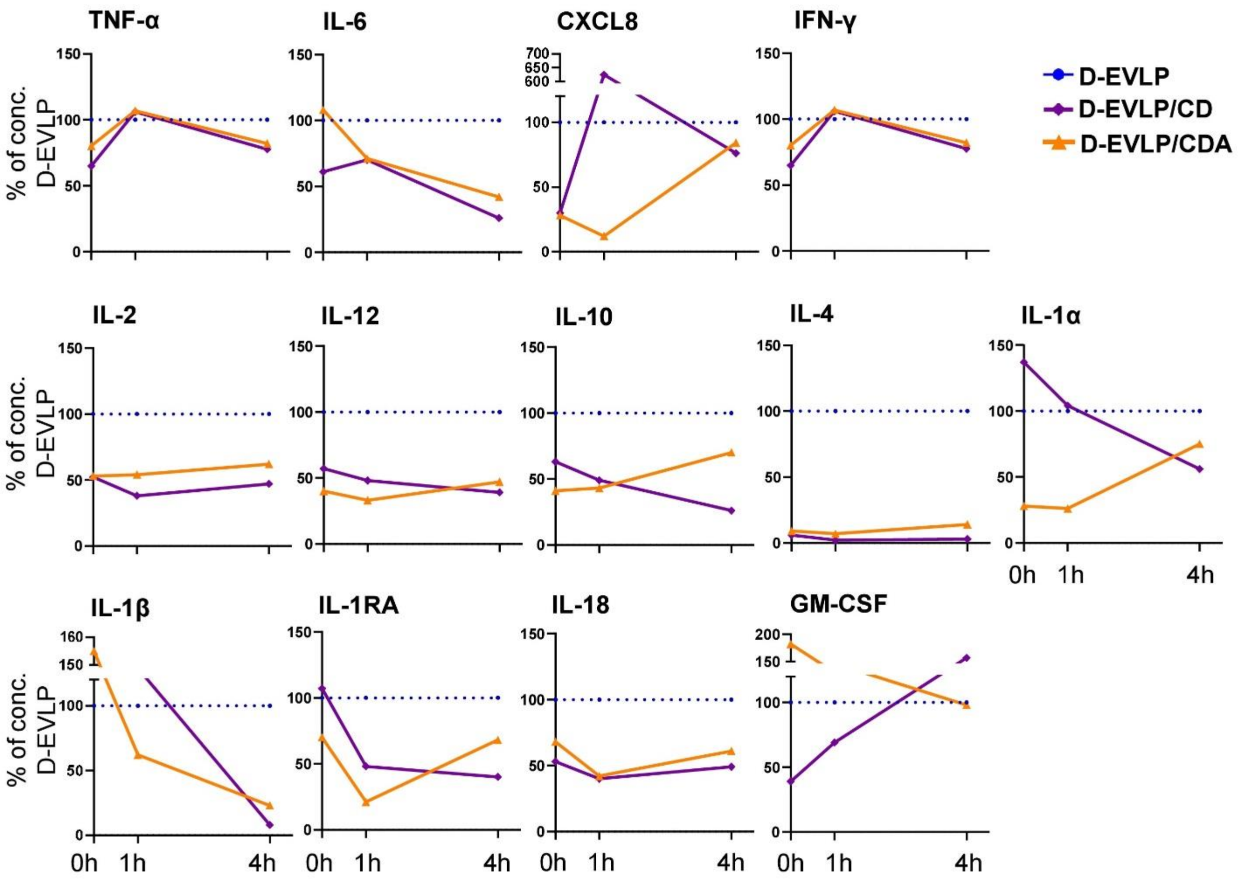

Supplementary Figure 3. Relative comparisons of delayed perfusion groups highlight decreased cytokine/ chemokine concentrations in the D-EVLP/CD and -/CDA groups**.** Perfusion samples for cytokine/chemokine quantification were obtained and measured as described in Fig.1. For each group and time point mean values were calculated. Mean values of the D-EVLP group were normalized to 100%. Concentrations of the D-EVLP/CD and -/CDA groups were displayed as % of D-EVLP. D-EVLP, delayed ex vivo perfusion with Steen solution; D-EVLP/CD, delayed ex vivo perfusion with Custodiol-N solution containing dextran; D-EVLP/CDA, delayed ex vivo perfusion with Custodiol-N solution containing dextran and albumin.

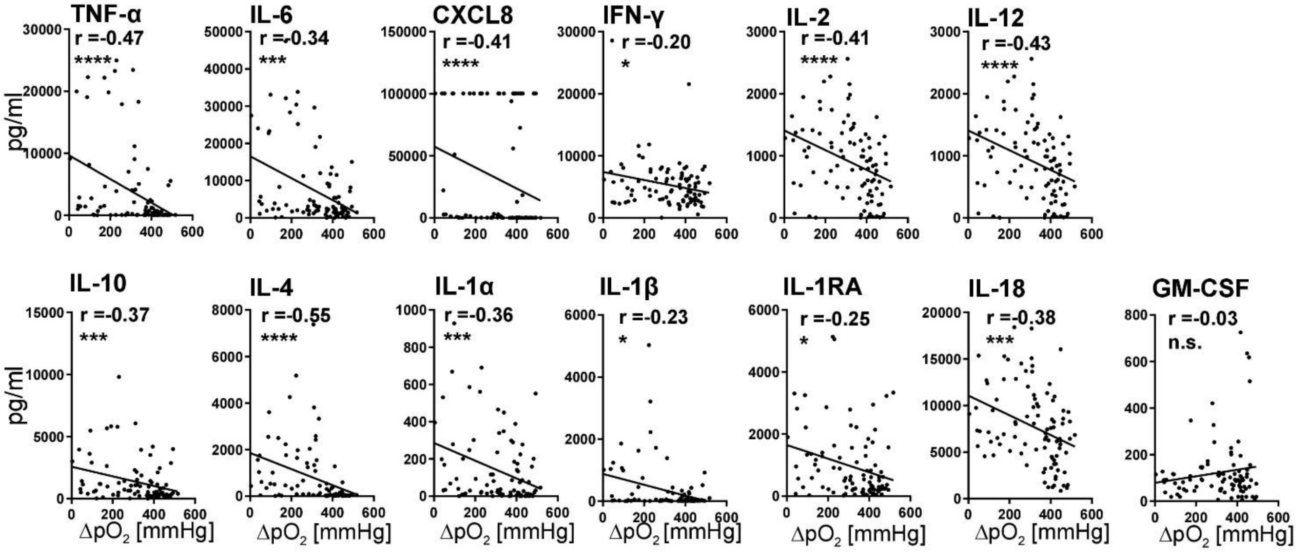

Supplementary Figure 4. Higher oxygenation capacities correlate with lower cytokine/chemokine concentrations in perfusates. Perfusion samples for cytokine/chemokine quantification were obtained and measured as described in Fig. 1. Oxygenation capacity (ΔpO2) was measured as described in the methods section. Correlation analyses (Spearman) of the oxygenation capacity and cytokine/ chemokine concentrations including all time points were calculated. To highlight the negative correlation between these two variables and linear regression of perfusion samples was performed. Asterisks indicate P values with *P < .05 **P < .01, ***P < .001, ****P < .0001.
